# A Prospective Clinical Study to Confirm the Safety and Performance of a Canister‐Based Single‐Use Negative Pressure Wound Therapy System in Low‐to‐Moderately Exuding Chronic Wounds

**DOI:** 10.1111/iwj.70983

**Published:** 2026-06-25

**Authors:** Berthold Amann, Michel Danneels, Joao Castro, Diletta Loschi, Mary‐Paula Colgan, Gomez Carlos Filipe, Vieira Nuna, Kerstin Kusch, Monira Nou Howaldt, Lorenzo Gibello, Fabio Verzini, Markus Stücker, Paulo Alves, Hilde Beele

**Affiliations:** ^1^ Franziskus‐Krankenhaus Berlin Germany; ^2^ AZ Delta Roeselare Belgium; ^3^ Unidade de Cuidados Continuados Integrados e Cuidados Palliativos Pavoa de Barzim Portugal; ^4^ Ospedale San Raffaele S.R.L. Milan Italy; ^5^ Rue da Mata Vila do Conde Portugal; ^6^ Complex Social da Moita Aveiro Portugal; ^7^ Unidade de Cuidados Continuadados Antonio Francisco Guimairaes Vizela Portugal; ^8^ Klinik fur Dermatologie, Venerologie und Allergalogie Berlin Germany; ^9^ CHU Montpelier Montpellier France; ^10^ AOU Citta della Salute e della Scienza di Torino Turin Italy; ^11^ Department of Dermatology Ruhr University Bochum Germany; ^12^ Ghent University Hospital Ghent Belgium

**Keywords:** chronic wounds, diabetes‐related foot ulcers, pressure ulcers, single‐use negative pressure wound therapy, venous leg ulcers

## Abstract

This clinical investigation was undertaken to confirm the safety and performance of a canister‐based single‐use negative pressure wound therapy system when used as intended in the management of low‐to‐moderately exuding chronic wounds, including diabetes‐related foot ulcers (*n* = 34), venous leg ulcers (*n* = 34), and pressure ulcers (*n* = 36) for up to 28 days. Using a prospective non‐comparative, multi‐centre design, device performance was evaluated at 14 sites in seven European countries. The study covered a broad spectrum of wounds, reflecting the inclusion of both inpatients (67.7%) and outpatients (32.3%). The primary outcome measure was wound progress. Overall, wounds were deemed improved since the previous clinic visit in 76.3% of visits and reductions in both absolute and relative wound size were noted over the study period. The device was considered effective and easy to apply by investigators, with no unexpected safety concerns noted.

## Key Points

- This prospective, multicentre European study evaluated the safety and performance of a canister‐based sNPWT system in 104 patients with diabetes‐related foot ulcers, venous leg ulcers, or pressure ulcers.
- Wound improvement was observed at 76.3% of clinic visits, with reductions in both absolute and relative wound size over the 28‐day study period.
- Investigators rated the device as effective and easy to apply across a broad inpatient and outpatient population.

## 1 Introduction

Chronic wounds affect a growing number of people around the world, with significant impact on physical health, mental well‐being, and overall quality of life. Over 10 million US Medicare beneficiaries were impacted by chronic wounds in 2019, up from 8.2 million in 2014 [1]. In Europe, up to 2 million people suffer from chronic wounds annually [2]. Estimates indicate that 1%–2% of the world's population will experience a chronic wound over their lifetime [3], amounting to a significant socioeconomic burden.

Chronic wounds are heterogeneous in cause and nature, but the most common types include diabetes‐related foot ulcers (DFUs), venous leg ulcers (VLUs) and pressure ulcers (PUs) [3]. Venous leg ulcers are the most common, affecting 1.5% of individuals over 65 years of age [3]. VLUs result from long standing underlying venous system insufficiency/pathology, valve malfunction, and capillary damage which leads to fluid leaking into the interstitial spaces [4]. In a recent report from the UK, only 53% of VLUs healed in any given year, with the majority of patients suffering from a wound for 2 years or more [5].

DFUs develop in 19%–34% of individuals with diabetes during their lifetimes [6], and contribute to over 85% of non‐traumatic amputations worldwide [7]. They develop as a consequence of factors including diabetic neuropathy and peripheral neuropathy, with a global clinical and economic burden estimated to be in excess of $40 billion annually [8]. The prevalence of diabetes is projected to increase around the globe by 69% in developing countries and 20% in developed countries between 2010 and 2030 [8].

PUs are most prevalent in patients who are immobile or infirm [3]. In a recent meta‐analysis of global prevalence data, PU rates varied from 3.4% to 32.4% of long‐term care residents [9]. Patient factors and the skill of staff caring for patients both contribute to PU incidence. Costs associated with PUs make up 4% of the UK's National Health Service total expenditure in 2024; costs were an estimated £1.4 million every day [5, 10].

Negative pressure wound therapy (NPWT) is an established means for managing hard‐to‐heal chronic wounds including DFUs, VLUs, and PUs [11, 12, 13, 14, 15, 16]. NPWT involves the topical application of sub‐atmospheric pressure to the wound–dressing interface [11, 17], which is thought to accelerate the healing of chronic wounds through stimulation of local blood supply, reduction of oedema, promotion of granulation tissue [18], and the removal of excess exudate. Reductions in wound volume, depth, and treatment duration as well as achievement of complete closure, increased granulation, and reduced amputation rates have been reported for chronic wounds treated with NPWT [19, 20, 21]. In a systematic review and meta‐analysis comparing NPWT to standard wound dressing, Liu et al. found that DFUs treated with NPWT had a shorter healing time, a higher rate of complete healing, and fewer amputations versus standard dressings, and treatment was more cost effective [22]. Similar results have been demonstrated for chronic VLUs, with improved time to complete healing and cost savings reported for NPWT versus conventional wound care [23, 24, 25]. Despite being widely used, the effects of NPWT on pressure ulcer healing are less certain due to a lack of well‐controlled trials [26].

Although NPWT was originally developed for in‐hospital use, portable single‐use negative pressure wound therapy (suNPWT) systems are now often favoured due to their small size, light weight, and the portability conferred by mechanical or battery powered systems [4, 17, 27, 28, 29, 30]. Portability makes these devices especially suitable for ambulatory patients in post‐acute, outpatient, and community care settings, with potential for accelerated patient transition from hospital to home [28]. Single‐use NPWT systems are canister‐based or canister‐less in nature [17, 27, 31, 32], with canister‐based systems removing exudate from the dressing that is in contact with the wound. Canister‐based systems provide more stable delivery of constant negative pressure [32] due to a reduced likelihood of dressing saturation with exudate [28], resulting in improved skin health and reduced incidence of infection [33]. In contrast, canister‐less systems rely solely on the ability of the dressing to manage exudate (by absorption and evaporation), and may be associated with a reduction in negative pressure to the wound bed corresponding to the level of dressing saturation [28].

This clinical investigation was undertaken to confirm the safety and performance of a canister‐based suNPWT system when used as intended in the management of low‐to‐moderately exuding chronic wounds for up to 28 days.

## 2 Materials and Methods

### 2.1 Device

**FIGURE 1:**
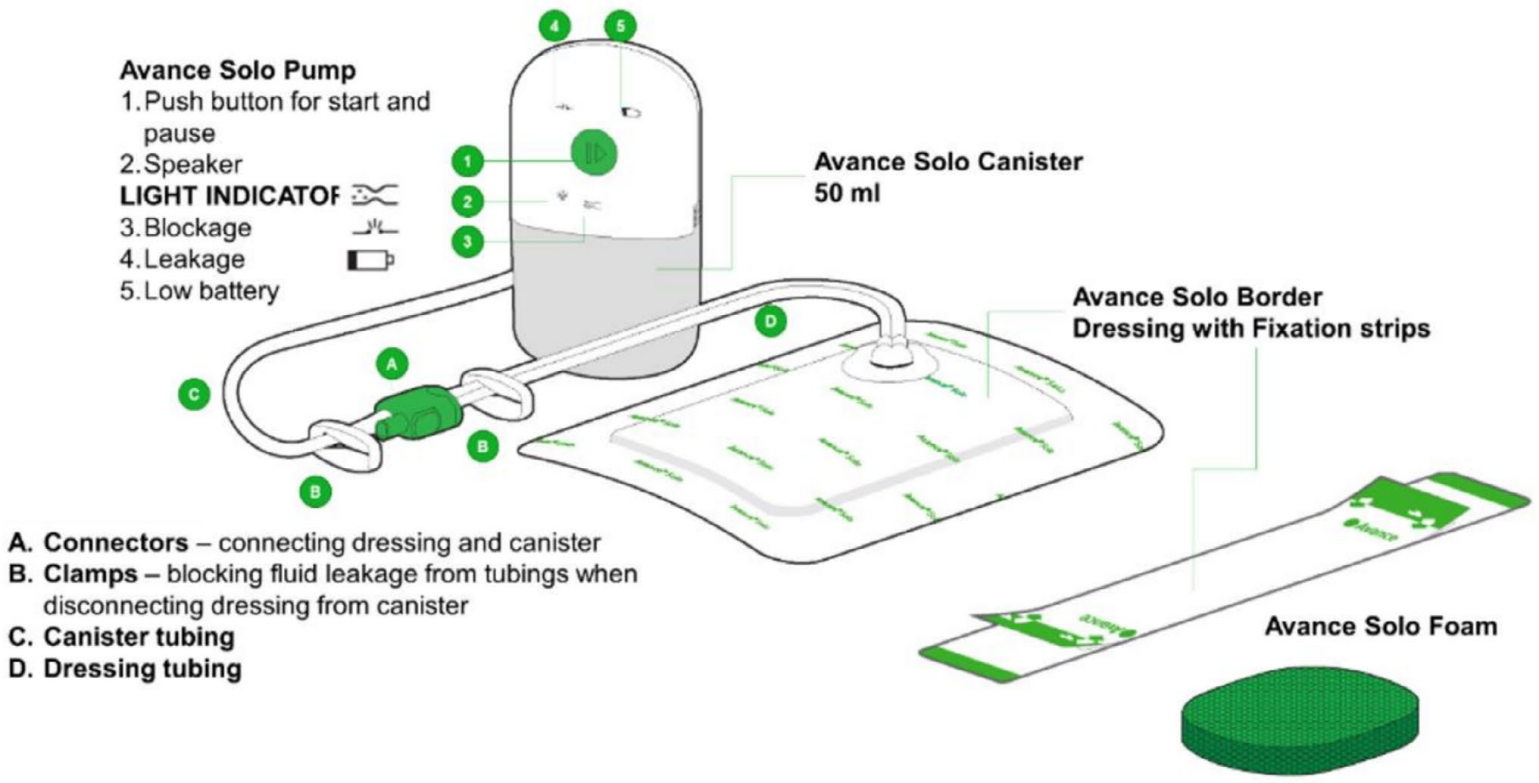
Schematic of the Avance Solo NPWT System.

The device investigated was a canister‐based suNPWT system (Avance Solo NPWT System; Mölnlycke Health Care, Mölndal, Sweden) which is a portable system incorporating a battery‐powered pump with a lifetime of 14 days. The device is supplied with a 50 mL collection canister for the removal of exudate from the supplied dressings of which there are two types: (1) Avance Solo Border Dressing (multi‐layered dressing; MLD) with fixation strips, and (2) Avance Solo Foam (used as a filler). Additional components include connectors, clamps, canister tubing, and dressing tubing (Figure 1). The Avance Solo NPWT system delivers a mean continuous negative pressure of −125 mmHg to the wound, and is indicated for use on wounds displaying low‐to‐moderate exudate levels. The MLD can be applied with or without the foam filler, depending on individual clinical characteristics.

### 2.2 Study Design and Participants

The study was designed as a prospective, open, non‐comparative, multicentre clinical investigation aimed at confirming the safety and performance attributes of a canister‐based suNPWT system when applied to low‐to‐moderately exuding chronic wounds in both in‐ and out‐patients for up to 28 days. Chronic wounds assessed included DFUs, VLUs, and PUs.

The study was conducted in accordance with the ethical principles of the Declaration of Helsinki and associated regulatory requirements, and was approved in writing by Research Ethics Committees at all institutions before enrolment of any subjects. The trial was registered at clinicaltrials.gov (NCT04753294), and followed ISO 141155 standards.

Informed consent was obtained, and prospective study participants were screened against study inclusion and exclusion criteria (Table 1) within 2 days of the baseline visit. At baseline, wound characteristics and demographic data were collected before the canister‐based suNPWT system was applied. Dressings were changed and wounds were evaluated every 7 days or earlier if needed, according to the device instructions for use (IFU) and standard practice at each study site. Foam as a wound filler was used if considered appropriate by the investigator in wounds with a depth greater than 0.5 cm. Where Avance Solo Foam was used, the dressing and foam were changed every 48–72 h (no less than 3 times a week), or as instructed by the investigator. At Day 14, a new Avance Solo NPWT system was applied.

**TABLE 1:** Study inclusion and exclusion criteria.

Inclusion criteria	Exclusion criteria
Male or female ≥ 18 years Signed written informed consent Low‐to‐moderate exuding chronic wounds (DFUs, VLUs, and PUs) suitable for suNPWT	Known malignancy in the wound or margins of the wound Untreated and previously confirmed osteomyelitis Non‐enteric and unexplored fistulas Presence of necrotic tissue with eschar Exposed nerves, arteries, veins, or organs Exposed anastomotic site Subjects with known allergies/hypersensitivity to product components Known pregnancy or planned to become pregnant, or lactation at time of clinical investigation participation Patients not suitable for the investigation according to the Investigator's judgement, clinical investigation plan and manufacturer's instructions for use (IFU) Current participation or participation in clinical investigations during the previous 30 days that could have had an impact on the outcome of the investigation based on the judgement of the investigator

### 2.3 Outcome Measures

**FIGURE 2:**
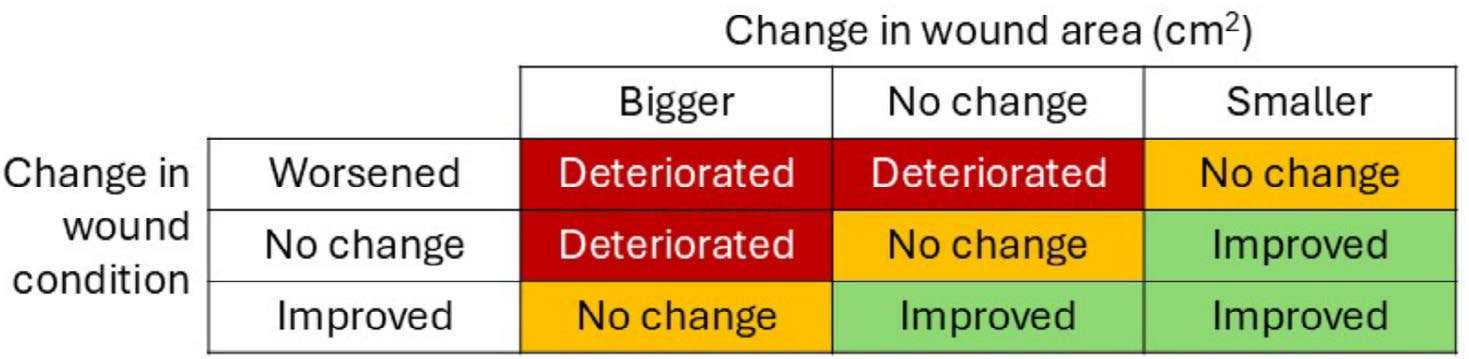
Standardized rubric for evaluation of the composite metric ‘wound progress’ used in this study. Wound progress was evaluated based on changes in wound area and condition between visits.

The primary endpoint was investigator‐assessed ‘wound progress’ compared with the previous visit for up to 28 days. To evaluate ‘wound progress’, investigators developed and used an evaluation rubric (Figure 2) incorporating both wound area (measured and recorded at each visit as bigger, no change, or smaller) and condition (peri‐wound skin condition, exudate, tissue type and pain level) (Figure 2). ‘Wound progress’ was defined as the intersection of both change in wound area and change in condition—for example, a wound that was reduced in size, but had worsened in condition would have been scored as ‘no change’, while a wound that was reduced in size and had improved in condition would be scored as ‘improved’ (Figure 2). To ensure that this measure was standardized across sites and visits, clinicians were trained on the tool at the outset of the study, and used photographs from the previous visit to score the wound condition as ‘worsened’, ‘no change’, or ‘improved’ since the previous visit. Standard wound measurements were taken at all visits and used to evaluate change in wound size since the previous visit. This comprehensive metric was intended to capture heterogeneous wound characteristics together as an overall indicator of wound progression towards healing.

Secondary endpoints assessed included wound progress between baseline and final visit; absolute and percentage change in wound area and volume; change in tissue type (slough, necrotic tissue, granulation tissue or epithelialisation), change in exudate amount (none, low, moderate, high), nature (serous, fibrinous, serosanguinous, sanguineous, seropurulent, purulent, foul purulent, haemopurulent, haemorrhagic) and odour (no odour, slight, moderate, strong, very strong); change in peri‐wound skin condition (normal, erythematous, oedematous, eczematous, excoriated, macerated, indurated); pain at dressing change, graded on a 10 point Numeric Rating Scale (NRS) from 0 (‘no pain’) to 10 (‘worst imaginable pain’). Investigators' and participants' global satisfaction regarding the device was evaluated at Day 28, or the final visit if treatment was discontinued early.

Safety endpoints were recorded including adverse events (AE) and serious adverse events (SAE).

### 2.4 Statistical Methods

#### 2.4.1 Determination of Sample Size

To reach a power of 80% for discrete and continuous variables, 34 subjects were required per indication (DFU, VLU, and PU), giving a total requirement of 102 patients.

#### 2.4.2 Statistical Evaluation

The main statistical analysis was undertaken on the full analysis set (FAS) which included all patients fulfilling all the inclusion/exclusion criteria and who had undergone at least one follow‐up measurement. Each wound subgroup was analysed separately and in an aggregated analysis, which combined all subgroups (combined group).

## 3 Results

A total of 104 patients were recruited to the study, conducted at 14 sites in seven European countries, including Belgium, Denmark, France, Germany, Italy, Portugal, and Ireland. Of the 104 eligible participants, 34 had a DFU, 34 had a VLU, and 36 had a PU. The study covered a broad spectrum of wounds, reflecting the inclusion of both inpatients (67.7%) and outpatients (32.3%) in the investigation. The average duration of wounds prior to study enrolment was 9.6 months, but VLUs had a significantly longer duration (15.4 ± 27.7 months) compared to DFUs (9.8 ± 20.9 months) and PUs (3.9 ± 4.2 months). Patient demographics and wound characteristics at baseline are described in Table 2.

**TABLE 2:** Patient characteristics at study initiation.

	Diabetes‐related foot ulcer (*n* = 34)	Venous leg ulcer (*n* = 33)	Pressure ulcer (*n* = 32)	Combined group (*n* = 99)
Gender				
Male	27/34 (79.4%)	15/33 (45.5%)	23/32 (71.9%)	65/99 (65.7%)
Female	7/34 (20.6%)	18/33 (54.5%)	9/32 (28.1%)	34/99 (34.3%)
Age	66.4 ± 13.1	74.9 ± 12.6	74.2 ± 13.7	71.8 ± 13.6
Coronary artery disease	12/34 (35.3%)	9/33 (27.3%)	12/32 (37.5%)	33/99 (33.3%)
Diabetes mellitus				
Type I	4/34 (11.8%)	0/33 (0.0%)	1/31 (3.2%)	5/98 (5.1%)
Type II	30/34 (88.2%)	6/33 (18.2%)	8/31 (25.8%)	44/98 (44.9%)
Peripheral artery disease	18/34 (52.9%)	11/33 (33.3%)	9/32 (28.1%)	33/99 (38.4%)
Abnormal renal function				
High	2/34 (5.9%)	0/33 (0.0%)	3/32 (9.4%)	5/99 (5.1%)
Low	11/34 (32.4%)	2/33 (6.1%)	8/32 (25.0%)	21/99 (21.2%)
Malnutrition	2/34 (5.9%)	2/33 (6.1%)	6/32 (18.8%)	10/99 (10.1%)
Mobility				
Ambulant	29/34 (85.3%)	32/33 (97.0%)	16/32 (50.0%)	77/99 (77.8%)
Bedridden	3/34 (8.8%)	1/33 (3.0%)	12/32 (37.5%)	16/99 (16.2%)
Chair bound	2/34 (5.9%)	0/33 (0.0%)	4/32 (12.5%)	6/99 (6.1%)
Setting of care				
Inpatient	21/34 (61.8%)	21/33 (63.6%)	25/32 (78.1%)	67/99 (67.7%)
Outpatient	13/34 (38.2%)	12/33 (36.4%)	7/32 (21.9%)	32/99 (32.3%)
Wound duration (months; mean ± SD)	9.8 ± 20.9	15.4 ± 27.7	3.9 ± 4.2	9.6 ± 20.4
Wound area (cm^2^; mean ± SD)	13.0 ± 21.6	17.5 ± 19.3	17.3 ± 24.7	15.9 ± 21.8
Wound volume (cm^3^; mean ± SD)	20.1 ± 30.9	9.1 ± 13.7	13.0 ± 19.6	13.9 ± 22.5
Wound cleansed	34/34 (100.0%)	30/33 (90.9%)	32/32 (100.0%)	96/99 (97.0%)
Debridement performed	14/34 (41.2%)	21/33 (63.3%)	18/32 (56.2%)	53/99 (53.5%)
No signs of local infection	31/34 (91%)	28/31 (90%)	26/32 (81%)	84/99 (85%)
Supporting treatment				
None	6/34 (18%)	3/33 (9.1%)	7/32 (22%)	16/99 (16%)
Compression	5/34 (15%)	29/33 (88%)	8/32 (25%)	42/99 (42%)
Off‐loading	24/34 (71%)	6/33 (18%)	23/32 (72%)	53/99 (54%)
Other	2/34 (5.9%)	1/33 (3.0%)	2/32 (6.2%)	5/99 (5.1%)

Those patients who had the canister‐based suNPWT applied at baseline and who went on to attend at least one follow‐up visit were included in further analyses (99/104 [95.2%]).

### 3.1 Primary Endpoint

**FIGURE 3:**
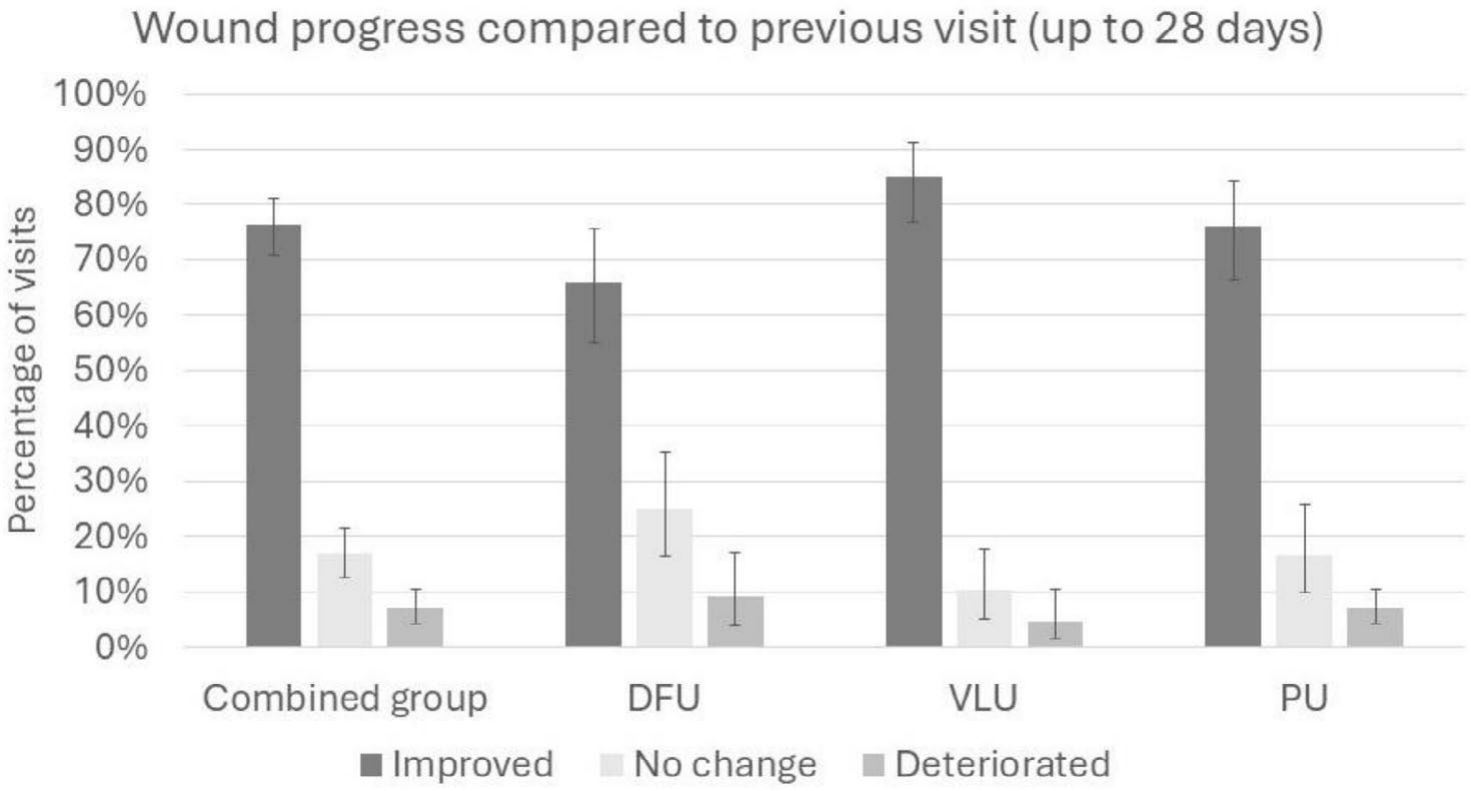
Wound progress according to standardized assessment methods over the 28‐day study period. 95% Confidence intervals (CI) shown. The lower limit of the 95% CI is above 50% and this result is therefore considered statistically significant.

The 99 patients attended a total of 291 follow‐up visits. The primary outcome was wound progress since the previous visit. This composite measure takes into account both wound area and wound condition (wound edge, wound bed, exudate level, and pain level), rated as a change since the previous visit on a pre‐specified standardized scale (Figure 2). Wounds were considered to have improved since the previous visit in 76.3% [95% confidence interval (CI) 71.0–81.1] of cases across all three wound types (Figure 3). DFUs showed improvement in 65.9% [95% CI 55–75.7] of visits, VLUs showed improvement in 85% [95% CI 76.9–91.2] of visits, and PUs showed improvement in 76.0% [95% CI 66.3–84.2] of visits.

### 3.2 Secondary Endpoints

#### 3.2.1 Change in Wound Area and Volume (From Baseline to Final Visit)

**FIGURE 4:**
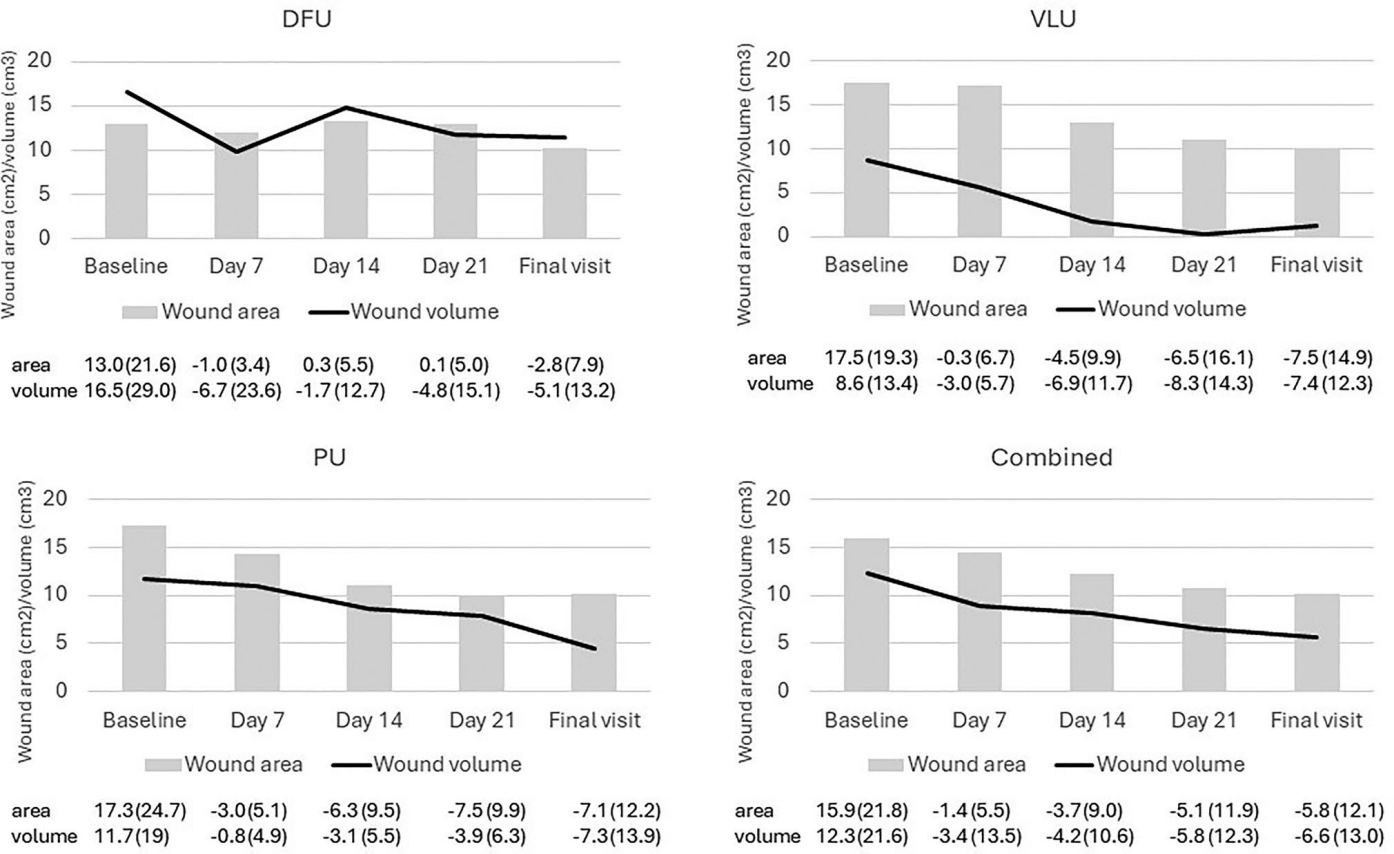
Change in wound area and volume from baseline to final visit by wound type and for all wounds combined. Wound measurements at baseline and change from baseline at each visit are included below the figures with standard deviations shown in brackets.

There was considerable variation in the area of wounds included at baseline, which resulted in some variability in response. Corresponding to improvements captured in our primary endpoint, decreases were noted in wound area (cm^2^) and volume (cm^3^) over the study period (Figure 4).

#### 3.2.2 Change in Tissue Type (From Baseline to All Follow‐Up Visits)

All tissue types were represented to some degree at baseline (necrotic, sloughy, granulation, and epithelialisation tissue). In the combined analysis, sloughy and necrotic tissue decreased throughout the study, and the degree of epithelialisation and granulation tissue increased. From baseline to final visit, the proportion of patients with no necrotic tissue increased by 23.6% and no sloughy tissue increased by 18.7%, respectively. Trends described for the combined population were reflective of each of the three wound subtypes (DFUs, VLUs, PUs).

#### 3.2.3 Change in Exudate Amount, Nature, and Odour (From Baseline to All Follow‐Up Visits)

For the combined group, the proportion of patients displaying no evidence of exudate increased from 1.0% at baseline to 14.6% at final visit. The proportion of patients with moderate exudate levels decreased by 21.5% compared with baseline (47.5% vs. 26.0%). Eleven participants (11.5%) were reported to have developed ‘high’ levels of exudate over the course of treatment, and NPWT was discontinued before the 28‐day timepoint in two of those participants. Generally, wound subgroups followed the combined group trends. At the final visit, 80/94 of investigators (85.2%) rated the system's ability to absorb and transport exudate as ‘good’ or ‘very good’.

Few changes in exudate nature were noted over the course of the study. At baseline, the proportion of patients with ‘no exudate’, ‘no odour’, or ‘slight odour’ was 1.0%, 56.6%, and 36.4%, respectively. At the final visit, 14.6% of participants had ‘no exudate’, 43.8% had ‘no odour’, and 24.0% had ‘slight odour’. However, there was an 8.5% increase in the proportion of patients with exudate of moderate odour at the final visit compared to baseline.

#### 3.2.4 Change in Peri‐Wound Skin Condition (From Baseline to All Follow‐Up Visits)

For the combined group, peri‐wound skin condition improved throughout the investigation, with an increase in the proportion of patients with normal peri‐wound skin condition (24.6% at baseline vs. 33.9% at final visit) and a decrease in the proportion with erythematous (27.1% at baseline vs. 14.8% at final visit), oedematous (5.7% at baseline vs. 3.5% at final visit), eczematous (11.5% at baseline vs. 7.0% at final visit), and indurated (4.1% at baseline vs. 0.9% at final visit) skin across all four visits. However, the proportion of wounds with both maceration (21.3% at baseline vs. 32.2% at final visit) and excoriation (2.5% at baseline and 7.8% at final visit) increased over the course of the study. Similar trends were observed for all subgroups, with increased maceration evident in DFUs, VLUs, and PUs across the majority of visits, with a small decrease at Day 7 for DFUs and Day 21 for PUs.

### 3.3 Other Secondary Endpoints

Throughout the investigation pain levels at bordered dressing change were generally low, with the lowest average on the NRS scale being 0.9 ± 1.4 (out of 10) in the combined group at the final visit. The trends were similar for all wound subtypes. Across follow‐up visits, the use of pain medication ranged from 55.1% at baseline to 51.8% at the final visit for the combined group.

Single‐use NPWT was reported to be easy to use; to transport and absorb exudate well; and to provide a high level of investigator and patient satisfaction at the final visit (70.3% of subjects and 80.8% of investigators reported being ‘satisfied’ or ‘very satisfied’ with the product at final visit). Incidence of wound site trauma was only reported in 5 of 96 (5.2%) cases in all groups combined at the final visit; trauma to the surrounding skin was reported in 17.7% of cases. When using suNPWT with foam, extensive tissue in‐growth was only observed at one dressing change (0.8% [1/131]).

Mean wear times for the pump and dressings were 11.1 ± 4.2 days and 4.5 ± 2.2 days, respectively, with a total of 41.4% of pumps used for the 14 days recommended in the IFU. The device was well received by both investigators and patients, with investigators evaluating ease of application and removal of the device to be ‘easy’.

### 3.4 Safety Evaluation

Safety data for 104 patients consisted of a total of 31 AEs reported in relation to 28 patients; of these AEs, four included information for two different medical events. Twenty‐nine AEs were skin‐related (Table 3). Nine serious adverse events (SAEs) were reported over the course of the study, including one death that was unrelated to the study procedures. Seven of the remaining SAEs were deemed unrelated to the study device or procedure, with one SAE being possibly device‐related (Table 3).

**TABLE 3:** Summary of adverse events and serious adverse events during the study.

AEs	Erythema (*n* = 4) Fluid discharge (*n* = 1) Swelling/oedema (*n* = 1) Tissue breakdown (*n* = 1) Wound infection (*n* = 2)
Device‐related AEs	Erythema (*n* = 1, possibly related) Wound odour with fluid discharge (*n* = 2, possibly related) Wound abrasion (*n* = 1) Contact dermatitis (*n* = 2, causal) Skin burning sensation (*n* = 1, causal) Skin erosion (*n* = 2 possibly related, *n* = 1 probably related) Fluid discharge with maceration (*n* = 1, probably related) Fluid discharge with skin erosion (*n* = 1 probably related)
SAEs	Wound infection (*n* = 2) Respiratory distress and pseudomembranous colitis (*n*=1) Sudden worsening of general condition (*n*=1) Sepsis (*n* = 1) Myocardial infarction (*n* = 1) Worsening of target wound (*n*=1, possibly related) Urinary tract infection (*n* = 2)

## 4 Discussion

The global burden of chronic wounds has inspired innovation in wound care, including the recent development of portable systems to allow use of NPWT in ambulatory patients. NPWT promotes wound healing through the promotion of a moist wound healing environment, reducing bacterial burden, removing oedema, and stimulating angiogenesis and blood flow to wound margins [34]. Single‐use NPWT systems are comparable to traditional NPWT in terms of reduction of wound area, wound volume, and achievement of complete closure for both VLUs [35] and DFUs [36]. More well‐controlled studies are needed to establish the role of suNPWT systems in the treatment of PUs [26].

In this multi‐centre study of DFUs, VLUs, and PUs in seven European countries, wounds responded well to treatment with the suNPWT across all subgroups considered. Investigators reported wound improvement in 76.3% of cases compared to the previous visit, across all wound types combined. Taken as both absolute and relative measures, wound area and volume were reduced across all wound categories. Secondary indicators of wound healing such as reductions in sloughy and necrotic tissue and increases in granulation and epithelial tissue formation were noted over the 28‐day treatment period. Our findings are in line with published accounts [36] of NPWT effectiveness in chronic wound healing.

NPWT is thought to support wound healing by removing excess exudate and mechanically stimulating the wound environment [31, 34, 37]. In this study, investigators rated the system's ability to absorb and transport exudate as ‘good’ or ‘very good’ at the final visit in 80/94 (85.2%) of cases. Almost 15% of patients had no exudate at study end (a 13.6% change from baseline), and the proportion of patients with moderate exudate level decreased by 21.5% compared with baseline (47.5% vs. 26.0%). Interestingly, a small increase was noted in patients with high levels of exudate over the study period. By study end, 11 participants (*n* = 3 DFUs, *n* = 4 VLUs, *n* = 4 PUs) were noted to have high levels of exudate. Wound odour was noted to be ‘strong’ in 1 wound and ‘very strong’ in two wounds at study end, potentially indicating infection in a subset of wounds.

Increases in exudate may also have contributed to increases in maceration noted in some cases across all visits for DFUs and VLUs. DFUs were noted to be on hard‐to‐dress areas, which potentially resulted in more dressing leakage and increased maceration. For PUs, maceration increased between baseline and first follow‐up visit (Day 7) with a simultaneous increase in granulation tissue. Along with this increase there was also an increase in exudate with a serosanguineous nature, which may be considered normal during inflammatory and proliferation phases of healing [38].

Low pain levels at dressing change were reported by patients. Avance Solo dressings contain Safetac, a soft silicone wound‐contact layer that has a long track record of mitigating dressing‐related trauma [39]. In a large multi‐national survey of 3034 participants with a variety of wounds (including DFUs, VLUs and PUs) measuring pain before, during, and after dressing change on a visual analogue scale, dressing with Safetac were associated with significantly less wound‐associated pain than alternative dressings with traditional adhesives [40]. Consistent with these recognized benefits of Safetac dressings, no (or very little) trauma to the wound and surrounding skin was noted at dressing change, and the majority of both patients and investigators reported being ‘satisfied’ or ‘very satisfied’ with the suNPWT system. No unknown or unexpected safety concerns were raised over the 28‐day study period. Performance and safety endpoints demonstrated the efficient performance of the canister‐based suNPWT under investigation in the treatment of PUs, DFUs and VLUs.

### 4.1 Limitations

Wound progress is a subjective measure, intended to capture the overall wound condition as a single variable. As with any clinical opinion, it is based on the experience level of the clinician. To mitigate this subjectivity, a variety of secondary endpoints were also captured. Together, these overall assessments of wound healing offer an holistic view of the healing trajectory. A limitation of this approach is that we did not capture complete wound healing in our investigation, choosing instead to focus on the kick‐starting of the healing phase in highly chronic wounds. As a result, many wounds were still healing at the conclusion of our investigation.

This study involved recruitment of participants at 14 centres across several European countries. Although this design led to a good degree of regional distribution to increase the generalisability of findings, there was significant variability in wound size at baseline as a result. This variability likely led to some heterogeneity in observed treatment effect, with wounds of different sizes benefiting to different degrees.

Lastly, this study was non‐comparative in nature, and should be interpreted accordingly.

## 5 Conclusions

Our study indicates that, for the management of low‐to‐moderately exudating chronic wounds, canister‐based suNPWT is associated with favourable outcomes with respect to exudate management, and wound progression. Patients reported minimal pain at dressing changes. No concerns about its safety were raised.

## Ethics Statement

The study was conducted in accordance with the ethical principles of the Declaration of Helsinki and associated regulatory requirements, and was approved in writing by Research Ethics Committees at all institutions before enrolment of any subjects. The trial was registered at clinicaltrials.gov (NCT04753294), and followed ISO 141155 standards.

## Conflicts of Interest

This study was sponsored by Mölnlycke Health Care. The authors declare no conflicts of interest.

## Data Availability

The data that support the findings of this study are openly available in clinicaltrials.gov at https://clinicaltrials.gov/, reference number NCT04753294.
